# Retraction: Effects of microRNA-374 on proliferation, migration, invasion and apoptosis of human SCC cells by targeting Gadd45a through P53 signaling pathway

**DOI:** 10.1042/BSR-20170710_RET

**Published:** 2021-04-23

**Keywords:** Apoptosis, Gadd45a, MicroRNA-374, P53 signaling pathway, Proliferation, Squamous carcinoma cells

This Retraction follows an Expression of Concern relating to this article previously published by Portland Press.

This article is being retracted from *Bioscience Reports* at the request of the Editor-in-Chief and the Editorial Board following receipt of a notification from a reader, alerting the Editorial Board to images that have been duplicated (from within the same article and from other published articles) in Figures 5C, 5D, 8A, 8C and 10A.

The authors were contacted regarding the concerns raised and wished to correct the article, however given the extent of the issues raised, the Editorial Board stands by the decision to retract the article. The authors disagree with the Journal's decision to retract the article and state that the duplications are due to errors being made by the authors during the submission of the article.

